# A current and historical perspective on disparities in US childhood pneumococcal conjugate vaccine adherence and in rates of invasive pneumococcal disease: Considerations for the routinely-recommended, pediatric PCV dosing schedule in the United States

**DOI:** 10.1080/21645515.2015.1069452

**Published:** 2015-09-16

**Authors:** John M McLaughlin, Eric A Utt, Nina M Hill, Verna L Welch, Edward Power, Gregg C Sylvester

**Affiliations:** Pfizer Vaccines; New York, NY USA

**Keywords:** pneumococcal conjugate vaccine (PCV), adherence, coverage, dosing schedule, disparities, race, minorities, socioeconomic status, pneumococcal disease, 2+1, 3+1

## Abstract

Previous research has suggested that reducing the US 4-dose PCV13 schedule to a 3-dose schedule may provide cost savings, despite more childhood pneumococcal disease. The study also stressed that dose reduction should be coupled with improved PCV adherence, however, US PCV uptake has leveled-off since 2008. An estimated 24–36% of US children aged 5–19 months are already receiving a reduced PCV schedule (i.e., missing ≥1 dose). This raises a practical concern that, under a reduced, 3-dose schedule, a similar proportion of children may receive ≤2 doses. It is also unknown if a reduced, 3-dose PCV schedule in the United States will afford the same disease protection as 3-dose schedules used elsewhere, given lower US PCV adherence. Finally, more assurance is needed that, under a reduced schedule, racial, socioeconomic, and geographic disparities in PCV adherence will not correspond with disproportionately higher rates of pneumococcal disease among poor or minority children.

## Introduction

Since its introduction to US infants and toddlers in 2000, 7-valent pneumococcal conjugate vaccine (PCV7), which was licensed in the United States as a 4-dose schedule (a 3-dose primary series at 2, 4, and 6 months with a booster dose at 12–15 months, aptly named a “3+1” schedule)^1^ has virtually eliminated US childhood invasive pneumococcal disease (IPD) caused by the 7 pneumococcal serotypes contained in PCV7.^2-11^ The subsequent introduction of 13-valent PCV (PCV13) in 2010 added protection against an additional 6 pneumococcal serotypes that i) were not previously covered by PCV7 and ii) become increasingly prevalent after PCV7 was introduced.^8,10-20^ Similar to PCV7, the Centers for Disease Control and Prevention (CDC) Advisory Committee for Immunization Practice (ACIP) and the American Academy of Pediatricians (AAP) currently recommend PCV13 be given as a 4-dose schedule to infants and young children in the United States.^21^ This 4-dose, US Food and Drug Administration (FDA)-approved PCV schedule has been associated with remarkable declines in vaccine-type childhood pneumococcal disease^2-12,^^22-24^ and corresponding herd protection among older adults in the United States.^7,9,^^22,25^

In October 2013 and February 2014, ACIP discussed reducing the US 4-dose PCV13 schedule to a 3-dose schedule—a schedule not licensed by the FDA. This discussion stemmed, in part, from economic considerations.^26^ While a reduced, 3-dose schedule has been a success in other developed countries that have national immunization programs (NIPs) with high levels of adherence (defined as receiving the recommended number of PCV doses within recommended or defined time intervals), childhood PCV adherence rates in the United States are appreciably lower.^9,27-29^ In 2013, approximately 1 in 4 US children was missing at least 1 recommended dose of PCV at age 19 months,^29^ and PCV adherence in the United States still lags behind other routinely-recommended US childhood immunizations.^30^ Moreover, US PCV adherence has plateaued within the last 5 years,^27,29,^^31^ and racial, socioeconomic, and geographic disparities persist in PCV uptake^9,27,^^29^

### Objective

The overarching goal of this review is to provide a current and historical perspective on US childhood PCV adherence and incidence of pneumococcal disease—ultimately to inform policy decision-making regarding the routinely-recommended pediatric PCV dosing schedule in the United States.

### History of racial/ethnic, socioeconomic, and geographic disparities in pneumococcal disease in the United States

Before the introduction of the 4-dose PCV7 series in the United States, the IPD rate among black children was more than twice that of white children.^5,8,^^32-37^ Disparities among other racial groups, including higher IPD rates among Alaskan Native infants, have been documented as well.^12,38-40^ Other studies have shown that significant socioeconomic disparities (e.g., poverty level, household income, health insurance coverage, and wealth) in IPD also existed.^6,8^ In addition to racial/ethnic and socioeconomic disparities in IPD, disparities by geographic location in both vaccine-type IPD prior to the introduction of PCV7 and rates of non-PCV7 replacement disease have been reported. ^20^

Drastic reductions in PCV7-type pneumococcal disease in the United States under a 4-dose schedule have translated into substantial declines in racial/ethnic, socioeconomic, and geographic disparities that previously existed with respect to PCV7-type IPD.^5,6,^^8,20,^^37,40^ Although racial and socioeconomic disparities in PCV7-type IPD gradually disappeared after the introduction of PCV7,^5,6,^^8,20,^^36,37,^^40^ disparities in IPD caused by serotypes not included in PCV7 (which, in 2009, were predominately the 6 additional serotypes in PCV13) emerged.^6,8,^^20,36,^^40^

**Figure 1.**
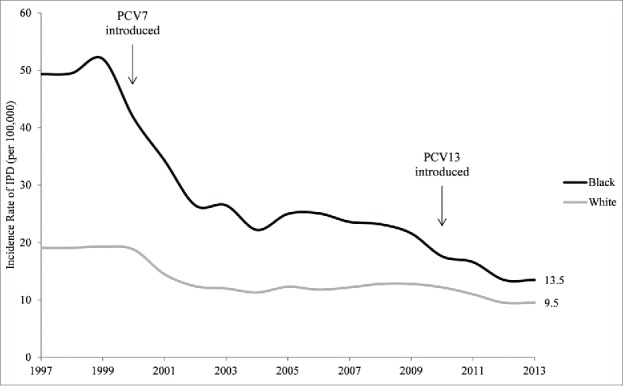
Invasive Pneumococcal Disease (IPD) Rates for Black and White US Persons (All Ages) Over Time, Active Bacterial Core Surveillance, 1997–2013.

Recent data have already shown substantial declines in PCV13-type IPD in the United States following the introduction of the 4-dose PCV13 series in 2010.^24,41^ Whether US rates of IPD (both vaccine-type and nonvaccine-type) among children differ by various racial, ethnic, socioeconomic, and geographic subpopulations today, in the *post*-PCV13 era, is unknown and should be continuously evaluated using CDC Active Bacterial Core Surveillance data. Recent CDC data, however, suggest that disparities in overall rates of IPD (both vaccine-type and non-vaccine type)—though drastically reduced by the introduction of PCV7 and PCV13 when administered as a 4-dose pediatric schedule—may still persist between white and black persons of all ages (**Fig. 1**).^9^

Continual monitoring of subpopulation-level differences in IPD and non-invasive pneumococcal disease will remain important, given that the same study that suggested a 3-dose schedule may be cost-effective^26^ also estimated that more disease would stem from a reduced dosing schedule.^26^ Much of the projected increase in disease burden under a reduced dosing schedule was comprised of non-invasive pneumococcal disease, particularly acute otitis media (AOM).^26^ This too may have implications for health disparities. Several studies have suggested that lower rates of AOM observed among poor and minority children are an under-representation due to differences in social determinants of health, such as access to care, health-seeking behavior, and/or diagnosis bias.^42,43^ Stated simply, poor and minority children may be less likely to receive appropriate diagnosis and care for AOM in the United States,^42,43^ and an increase in AOM stemming from a reduced dosing schedule^26^ could exaggerate this effect.

Today, it is not known whether poor and minority children, who have historically been at much greater risk for pneumococcal disease, would bear a disproportionate burden of this predicted uptick in disease^26^ should a reduced dosing schedule result in diminished protection. While we should continue to monitor disparities in both vaccine-type and nonvaccine-type IPD moving forward, we should also acknowledge the remarkable accomplishment of the 4-dose PCV series, to date, in erasing a long history of disproportionately higher vaccine-type IPD rates among several vulnerable subpopulations in the US. Root causes for historical differences in burden of pneumococcal disease by racial/ethnic, socioeconomic, and geographic subpopulations are complex, including differences in rates of influenza, child day care attendance, crowding, air pollution, breast feeding practices, prevalence of underlying comorbid disease (e.g., asthma, sickle cell, or HIV/AIDS) and other risk factors like exposure to second-hand smoke, health insurance coverage, and general access to health care.^6,8,^^20,36,^^44^ Another potential explanation for disparities in vaccine-type IPD is that appreciable differences persist in PCV adherence across certain racial/ethnic, socioeconomic, and geographic subpopulations in the United States.^24^

## PCV adherence in the United States

**Figure 2.**
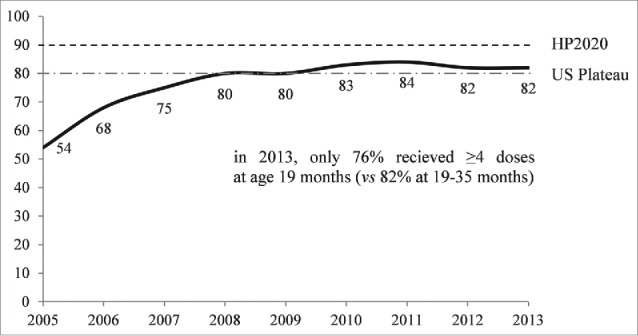
Percentage of US children who Received ≥4 Doses of PCV by Age 19–35 Months, National Immunization Survey, 2005–2013.CDC/NCHS and National Center for Immunization and Respiratory Diseases, National Immunization Survey. Available from: http://www.cdc.gov/vaccines/stats-surv/imz-coverage.htm#nis and http://www.cdc.gov/nchs/nis.htm. See Appendix I, National Immunization Survey (NIS). Table 78.CDC. National, State, and Selected Local Area Vaccination Coverage among Children Aged 19–35 Months — United States, 2013. *MMWR*. Weekly / Vol. 63 / No. 34. August 29, 2014.

PCV adherence is a priority of Healthy People (HP) 2020, with a goal of 90% 4-dose PCV uptake for children aged 19–35 months.^45^ The most recent (2013) CDC National Immunization Survey (NIS) estimates, however, show that 4-dose PCV adherence remains below the HP2020 target—with 82% of children aged 19–35 months receiving all 4 recommended doses (92% of children aged 19–35 months received ≥3 doses).^29^ NIS data also suggest that US 4-dose PCV adherence levels have plateaued near 80% for that age group since 2008 (**Fig. 2**). ^27,29,^^31^

**Figure 3.**
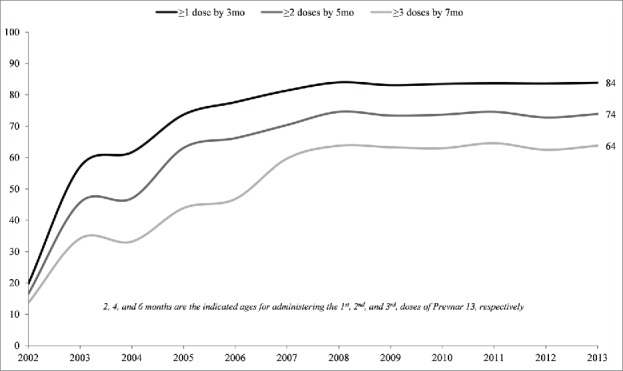
Percentage of US Children who Received ≥1, ≥2, and ≥3 Doses of PCV by Age 3, 5, and 7 Months, respectively, National Immunization Survey, 2002–2013CDC/NCHS and National Center for Immunization and Respiratory Diseases, National Immunization Survey, Coverage Level by Milestones. Available from: http://www.cdc.gov/vaccines/imz-managers/coverage/nis/child/index.html.

NIS PCV milestone adherence rates are slightly lower than the standard CDC time window of 19–35 months. In 2013, approximately 1 in 4 US children was missing at least 1 dose of PCV at age 19 months, and 1 in 5 were missing at least 1 dose at age 24 months.^29^ This is lower than PCV adherence levels obtained in other developed countries.^28,46,^^47^ For example, in 2013, in the United Kingdom, 95% of infants and young children had completed the recommended primary series of PCV by age 12 months, versus only 87% in the United States by age 13 months. Similarly, in the United Kingdom, 93% of children had received their PCV booster by age 24 months, whereas 4-dose PCV adherence rates in the United States were only 80% by age 24 months.^28,29^ NIS data also show that US PCV adherence decreases notably at each subsequent primary series milestone, with more than 1 in 3 children missing at least one primary series dose at age 7 months (**Fig. 3**).^31^ Among infants and children aged 5–19 months, an estimated 24–36% are essentially already receiving a reduced PCV schedule (i.e., missing at least one dose) in the United States.^27-31^ By 2 y of age, 20% of US children are still missing 1 or more recommended doses of PCV.^31^ This raises a practical concern that, under a reduced (3-dose) schedule in the United States, a similar proportion of young children may receive only 2 or fewer doses.

### Differences in PCV adherence by race, socioeconomic status, setting of care, and geographic region

Recent CDC NIS data show that PCV adherence differs by racial/ethnic, socioeconomic, and geographic subpopulations. In 2013, among children aged 19–35 months, there were statistically significant differences in the percentage of children who received ≥4 PCV doses by race (white: 84% *vs* black: 76%, p<.05) and by federal poverty level (FPL) (≥FPL: 86% *vs* <FPL: 75%, p<.05).^29^ Racial differences were diminished after statistical adjustment for poverty status—suggesting that socioeconomic factors are likely the driving force behind racial differences in immunization rates (**Table 1**).^27,29^ US Census data support this, showing that 41% of all black children and 32% of Hispanic children aged younger than 5 y live below the poverty level, compared with only 13% of white children.^48^ Poverty seems to be the primary driver for PCV adherence and affects all races indiscriminately, with differences in PCV adherence by poverty level for both white and black children (**Table 1**).

**Table 1.** Percent of US Children aged 19–35 months who received ≥4 doses of PCV by federal poverty level and racial group, 2013 National Immunization Survey

Racial Group			
Federal Poverty Level (FPL)	White Children	Black Children	All Races
≥FPL	87.5%	79.8%	86.1%
<FPL	71.7%	71.8%	74.5%

Federal poverty level (FPL) depends on the number of individuals in a family or household. In 2014, for a one-person household, the FPL was $11,670 for the 48 Contiguous States and the District of Columbia (FPL is slightly higher in Alaska and Hawaii). For each additional family/household member, FPL increases by $4,060 (e.g., FPL is $23,850 for a 4-person household).

Differences in PCV adherence also exist by the type of health care setting in which PCV is administered.^27,29^ Among children aged 19–35 months, the percentage of children who received ≥4 doses of PCV in the private setting (e.g., private practices/clinics, HMOs, and group practices) was 85% in 2013, as opposed to 75% for public providers (i.e., public health clinics and community health centers).^29^ It is likely that socioeconomic status is also related to the type of health care setting in which immunizations are received, given that public health clinics and community health centers tend to serve a large proportion of uninsured and low-income populations. Vaccination rates also varied significantly by geographic region with Arkansas having the lowest PCV uptake (only 70% of children aged 19–35 months received ≥4 doses of PCV in 2013) compared to other geographic areas.^29^

### The need for a renewed focus on pediatric PCV adherence

Historically, disparities in childhood immunization rates have been reported in the United States,^49-51^ with lower rates among children living in poverty,^52-54^ among urban children,^55^ and among black and Hispanic children.^51,56-59^ While Medicaid, the Children's Health Insurance Program (CHIP), and other federally-funded programs such as Vaccines for Children (VFC) have made significant strides in narrowing disparities associated with vaccine uptake and access to vaccines,^60,61^ differences persist for PCV adherence.^27^ Particular attention needs to be paid to areas of historically low immunization rates in which children are also at significantly greater risk of vaccine-preventable diseases.^55-59,^^62,63^ These areas, coined “pockets of need” by the US General Accounting Office,^64^ are typically urban, underserved areas, characterized by crowding, poverty, and inadequate health care utilization, which can act together to simultaneously produce low immunization rates and raise disease risk.^65^ Indeed, it is critical to ensure that a window of exposure (i.e., an inadequate number of or a longer gap between PCV doses) not be opened further for the most vulnerable children in our nation who are already at increased risk for developing pneumococcal disease.^6,8,^^9,24,^^27^ The diversity of the US population, both racially and socioeconomically, combined with patient-, provider-, and US health care system-level challenges in obtaining and maintaining high levels of PCV adherence for all US children, calls for a renewed focus on addressing existing disparities in PCV uptake.

Recent quality improvement studies have demonstrated that, in today's health care environment, outreach and care coordination activities are most successful at bringing under-immunized children up to date and that multicomponent interventions—specifically those that identify community-wide partners—are needed for improving immunization rates in socioeconomically disadvantaged populations.^66,67^ There is also a pervasive concern among medical and public health experts that the number of parents who are refusing recommended vaccinations for their children is on the rise.^68-71^ Recent research has suggested that underscoring how parents' own children would benefit (vs. larger societal benefits) is key for persuading parents to follow recommended vaccination guidelines.^72^ Health disparities in vaccination rates and in historical rates of disease represent a complex interplay between biological and genetic, social and cultural, psychological and emotional, socioeconomic, geographic (e.g., crowding, air pollution, rural *vs* urban), and health care system-related factors. These factors, either alone or in combination, are the building blocks for the construction of health disparities. Identifying which of these factors are i) most easily modifiable and ii) could best be targeted by health interventions is paramount for improving access to care and health outcomes among subpopulations at increased risk for disease.

## Conclusions

The 4-dose, FDA-approved PCV schedule has been associated with remarkable declines in vaccine-type childhood pneumococcal disease^2-12,^^22-24^ and corresponding herd protection among older adults in the United States.^7,9,^^22,25^ In October 2013 and February 2014, ACIP discussed reducing the US 4-dose PCV schedule to a 3-dose schedule—a schedule not licensed by the FDA. Recent CDC data,^27,29,^^31^ however, suggest that PCV adherence rates in the United States remain below US HP2020 goals and below that of other countries.^28,29,^^31^ Thus, we should increase efforts to narrow the gap in adherence to a public health intervention shown to prevent childhood morbidity and mortality and to provide substantial medical and societal cost savings.^73,74^ Public health officials and policy makers should consider targeted and tailored state- and community-based strategies for improving PCV adherence and achieving HP2020 goals, especially in subpopulations with historically low immunization rates and significantly greater risk of vaccine-preventable diseases. Among infants and children aged 5–19 months, an estimated 24–36% are already receiving a reduced PCV schedule (i.e., missing at least one dose) in the United States.^27,29,^^31^ This raises a practical concern that, under a reduced (3-dose) schedule, a similar proportion of young children may receive only 2 or fewer doses.

Based on 2013 NIS data, 92% of children have received ≥3 doses of PCV in the United States. However, it is important to remember that 3-dose adherence achieved in the United States is achieved under a recommended *4-dose* schedule. Thus, it is unreasonable to expect that 3-dose adherence rates would remain the same under a reduced, 3-dose schedule as they are today under the currently recommended, 4-dose schedule. As such, comparing 3-dose US adherence data (when the 4-dose series is licensed and recommended) to 3-dose adherence data in other countries where only 3 doses are recommended is likely a biased comparison.

A CDC-sponsored cost-effectiveness study previously stressed that, to prevent additional pneumococcal disease, any PCV dose reduction should be coupled with a significant increase in PCV adherence.^26^ This suggestion, however, appears unrealistic in the near-term given that PCV adherence rates have leveled-off since 2008.^9^ Furthermore, increasing PCV vaccination rates on a national scale would require significant economic investment, and these additional costs were not accounted for in the cost-effectiveness analysis. At the same time, there is new evidence that suggests levels of parental vaccine hesitancy may be on the rise,^68-71^ fueled, in part, by recent enhancements of the anti-vaccine platform on the Internet and social media.^75^ This increasing substandard vaccination adherence is not without consequence, and, most recently, was likely responsible for the 2015 measles outbreak.^76^

In addition, is it reasonable to expect that a reduced, 3-dose PCV schedule in the United States will afford the same protection against pneumococcal disease as 3-dose schedules used elsewhere in the developed world, given lower PCV adherence rates observed in the United States?^28,29^ This uncertainty is compounded by the fact that recent data from the United Kingdom, where only 3 doses (rather than 4) of PCV13 are routinely given, suggest that while 3 doses of PCV may have been sufficient to obtain protection against PCV7-type disease, >3 doses may be needed to ensure protection against some PCV13 serotypes—notably 19A.^77^ Further, a notably large proportion of breakthrough cases of IPD observed in the United Kingdom occurred among children aged 6–12 months—the time period during which a primary dose was removed (prior to receiving a booster) in a reduced, 2+1 schedule.^77^

Finally, the risk of increased disease exposure, as was predicted by Stoecker et al.^26^ must be considered in any reduction of dosing schedule. More research is needed into the underlying causes of disproportionately lower PCV adherence among certain racial/ethnic, socioeconomic, and geographic subpopulations. Most importantly, more assurance is needed that, under a reduced schedule, these racial, socioeconomic, and geographic disparities in PCV adherence^27,29^ will not correspond with disproportionately higher rates of pneumococcal disease among poor or minority children. This consideration, especially, remains a dilemma for both the policymaker and clinician.

## Disclosure of Potential Conflicts of Interest

Drs. McLaughlin, Utt, Hill, Welch, Power, and Sylvester are employees and shareholders of Pfizer Inc.
